# Squamous cell carcinoma of ascending colon with pMMR/MSS showed a partial response to PD-1 blockade combined with chemotherapy: A case report

**DOI:** 10.3389/fonc.2023.1051786

**Published:** 2023-03-09

**Authors:** Yan Liu, Junliang Du, Pan Zhang, Wenjuan Meng, Haifeng Xiao

**Affiliations:** Oncology Department, Weifang People’s Hospital, Weifang Medical University, Weifang, China

**Keywords:** ascending colon, squamous cell carcinoma, immunotherapy, chemotherapy, pMMR/MSS, BRAF V600E, PD-L1

## Abstract

Primary colon squamous cell carcinoma (SCC) is extremely rare and associated with a poor prognosis. Moreover, there is no treatment guideline for this disease. Proficient mismatch repair/microsatellite-stable (pMMR/MSS) colorectal adenocarcinoma is refractory to immune monotherapy. Although the combination of immunotherapy with chemotherapy in pMMR/MSS colorectal cancer (CRC) is currently under investigation, the clinical activity of this approach in colorectal SCC remains unknown. In this article, we report the case of a pMMR/MSS CRC patient with ascending colon SCC who had high programmed cell death–ligand 1 (PD-L1) expression and the a missense mutation in codon 600 of the B-Raf proto-oncogene (BRAF ^V600E^) mutation. The patient exhibited a significant response to the combination of immunotherapy and chemotherapy. After eight cycles of treatment with the combination of sintilimab and mFOLFOX6 (oxaliplatin, fluorouracil, and leucovorin), computed tomography–guided microwave ablation of the liver metastasis was performed. The patient achieved excellent durable response and continues to experience a good quality of life. The present case indicates that programmed cell death 1 blockade combined with chemotherapy may be an effective therapy for patients with pMMR/MSS colon SCC and high PD-L1 expression. Furthermore, PD-L1 expression may be a biomarker for immunotherapy in patients with colorectal SCC.

## Introduction

Primary squamous cell carcinoma (SCC) of the colon is an extremely rare type of tumor; this disease accounts for 0.1%–2.5% of all colorectal malignancies (1). Owing to the rarity of the disease, a treatment guideline for colon SCC has not been established thus far. The current optimal treatment is surgical excision of the primary tumor, followed by adjuvant chemoradiotherapy. The use of immunotherapy in colon SCC is infrequently reported. In this article, we report the case of a patient with proficient mismatch repair/microsatellite-stable (pMMR/MSS) colon SCC who had high programmed cell death-ligand 1 (PD-L1) expression and the BRAF^V600E^ mutation. The patient showed significant response to the combination of programmed cell death 1 (PD-1) blockade and chemotherapy.

## Case report

A 76-year-old female patient was admitted to hospital for lower abdominal intermittent pain and melena in November 2021. An obstructing mass in the ascending colon was detected through colonoscopy, and histopathology analysis confirmed the presence of poorly differentiated carcinoma. Abdomen–pelvis computed tomography (CT) revealed a large obstructing mass in the ascending colon invading the intestinal wall, accompanied by mesenteric lymphadenectasis in multiple lymph nodes and multiple liver metastases. Further examination through ^18^F-fluorodeoxyglucose positron emission tomography/CT (PET/CT) showed hypermetabolism and segmental eccentric thickening of the ascending colon wall, which is consistent with colon cancer. The enlarged mesangial lymph nodes in the medial ascending colon, multiple intrahepatic nodules, and nodules in the right upper lobe with hypermetabolism were noted as metastases. The patient was diagnosed with primary colon SCC (cT4aN2aM1b, stage IV), accompanied by multiple liver and lung metastases.

The patient underwent a palliative right hemicolectomy and partial resection of the liver in November 2021. Histopathology demonstrated a poorly differentiated SCC of the ascending colon infiltrating the serosa. Metastatic lesions were found in lymph nodes (5/17). Poorly differentiated carcinoma was observed in the liver tissue; this finding is consistent with the liver metastasis of colon cancer. Immunohistochemistry (IHC) revealed the PD-L1 combined positive score (CPS) of 95, with pMMR/MSS status. Using the amplification refractory mutation system–polymerase chain reaction (ARMS-PCR), the BRAF^V600E^ mutation was detected, while the analysis did not reveal mutations in the KRAS and NRAS genes. This kit can test the 12/13/61 codons of the Kirsten rat sarcoma viral oncogene homolog (KRAS) and Neuroblastoma RAS viral oncogene homolog (NRAS) genes, as well as detect the BRAF^V600E^ mutation. After surgery, the patient refused to receive follow-up antitumor therapy due to poor physical condition.

**Figure 1:**
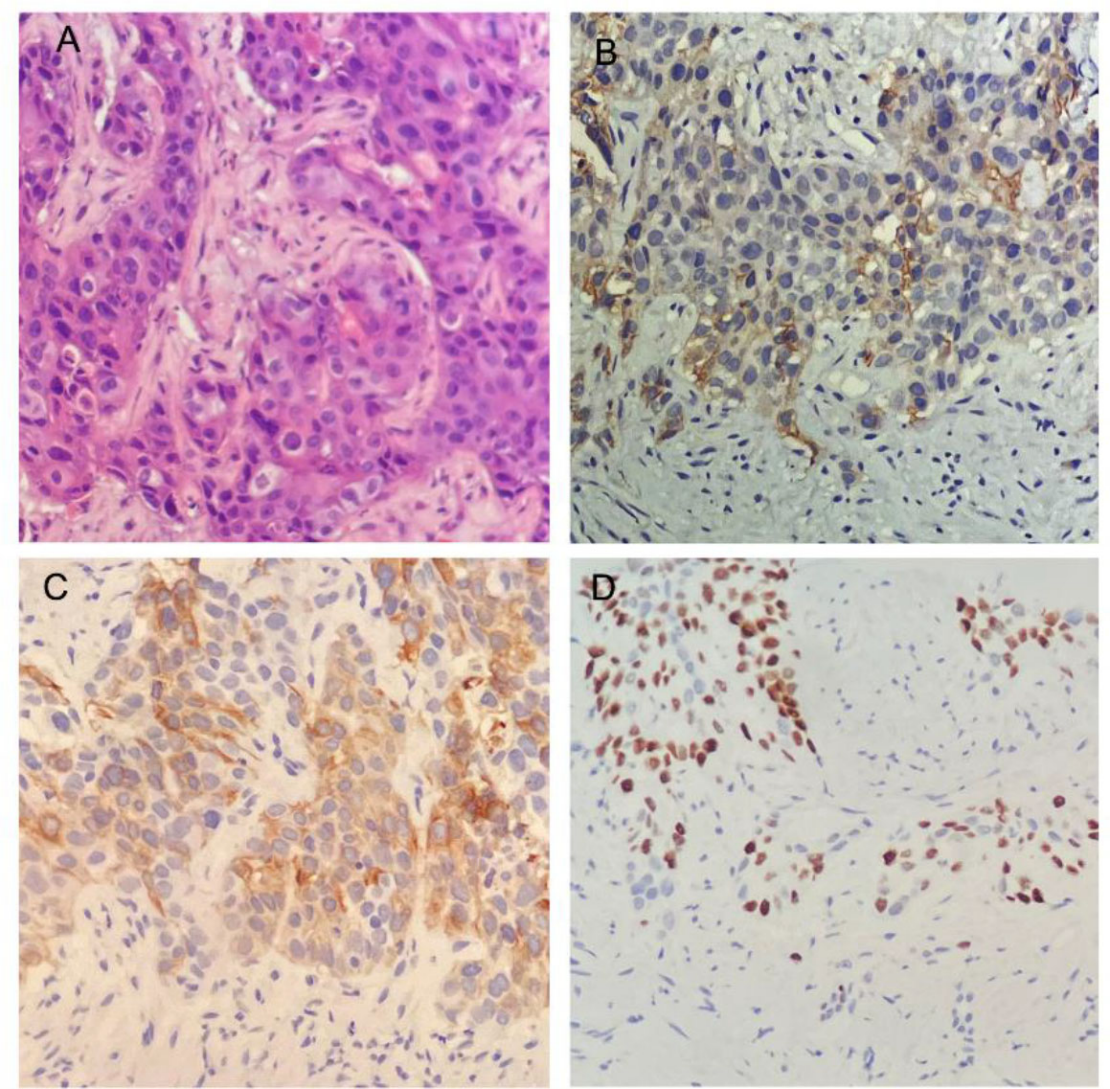
Histopathology and immunohistochemistry (IHC) of the biopsies from liver metastases. **(A)** Histological findings revealed squamous cell carcinoma (HE × 200). **(B)** IHC staining for the expression of programmed cell death–ligand 1 (×200). **(C, D)** IHC staining yielded positive results for CK20 **(C)** and P40 **(D)** (×200).

New lesions were detected in the liver by CT 3 months after operation, and the patient was admitted to our hospital in February 2022. The biopsy of the hepatic lesions revealed metastatic SCC (**Figure 1A**). The PD-L1 CPS was 20 (**Figure 1B**). The immunohistochemical staining of the biopsy specimen revealed a positive expression of CK20 (**Figure 1C**), P40 (**Figure 1D**), P63, CK20, CK5/6, MLH1, MSH2, MSH6, and PMS2. The expression of PD-L1 protein was evaluated with an IHC method using a mouse monoclonal antihuman PD-L1 antibody (Clone 22C3; Dako).

**Figure 2:**
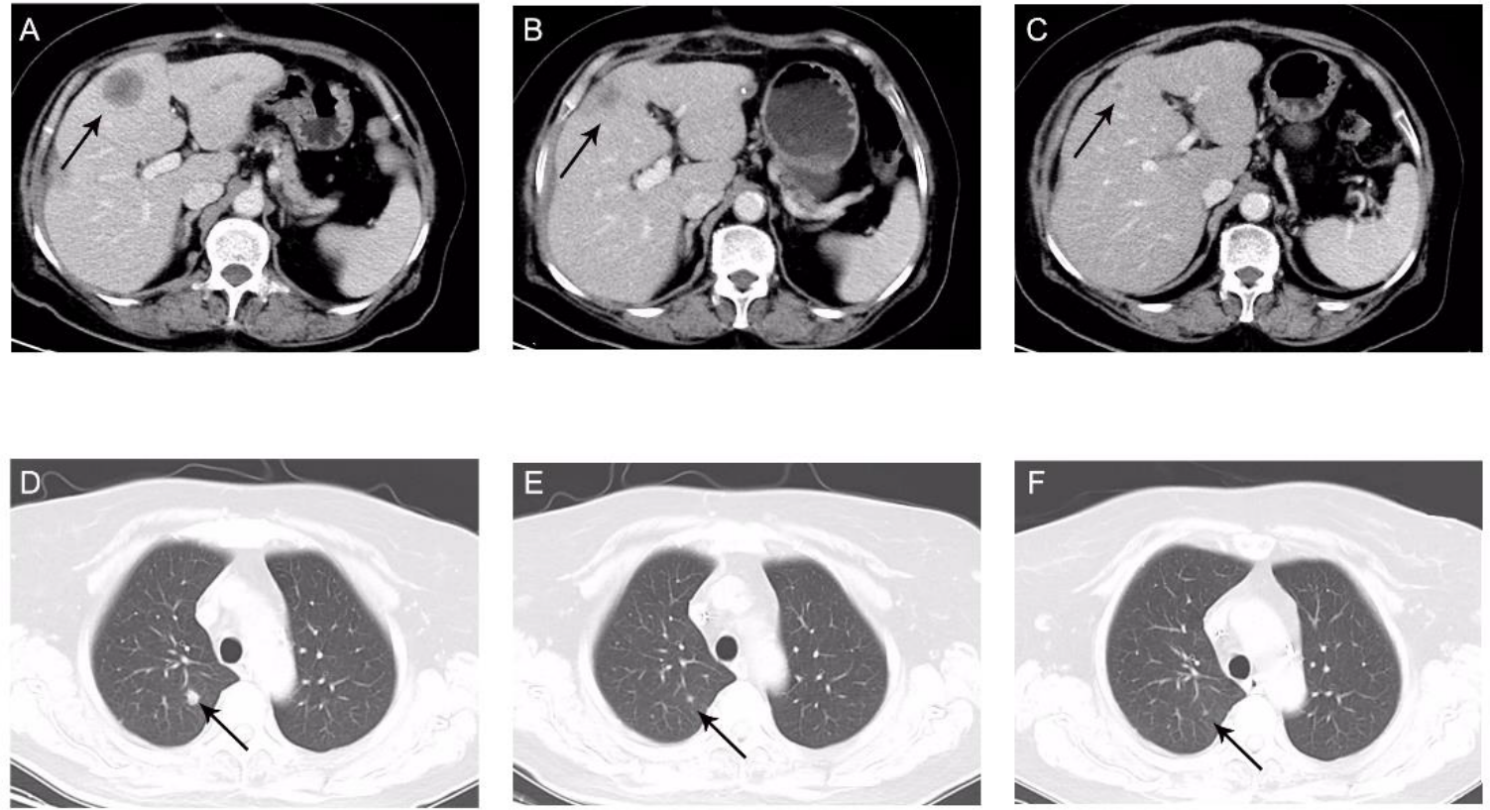
Computed tomography portal phase images. Maximum diameter of the liver **(A–C)** and lung **(D–F)** metastases. **(A)** 28.7 mm, before the combination therapy. **(B)** 18.46 mm, after two cycles of combination therapy. **(C)** 6.39 mm, after eight cycles of combination therapy. **(D)** Before combination therapy. **(E)** After two cycles of combination therapy. **(F)** After eight cycles of combination therapy.

Considering the poor physical condition of the patient and high expression of PD-L1, we administered immunotherapy with sintilimab (200 mg, once every 21 days (q21d)) plus chemotherapy with mFOLFOX6 (oxaliplatin, fluorouracil, and leucovorin). The mFOLFOX6 regimen consisted of oxaliplatin (120 mg intravenously guttae (ivgtt)), fluorouracil (600 mg iv, 3,625 mg civ 46 h), and leucovorin (600 mg ivgtt) and was administered every 14 days. After two cycles of combination therapy, CT showed that the liver lesion had shrunk from 28.7 to 18.46 mm (**Figures 2A, B**); hence, the patient had achieved a partial response. The liver lesion had shrunk to 6.39 mm after eight cycles of the combination therapy (**Figure 2C**). Significant tumor shrinkage was also observed in the lung lesion (**Figures 2D–F**). Importantly, there were no drug-related adverse events noted, except for grade 1 myelosuppression.

To improve the efficiency, the patient underwent CT-guided microwave ablation of liver metastases after eight cycles of the combined therapy. There were no residual lesions in the liver following the microwave ablation. Subsequently, monotherapy with sintilimab was administered once every 3 weeks. The patient continues to exhibit a stable response and experiences a good quality of life. In November 2022, the enlargement of retroperitoneal lymph nodes was discovered. Progression-free survival (PFS) was 8.5 months.

The patient provided informed consent for the publication of this case.

## Discussion

Colorectal SCC typically occurs in the fifth decade of life, and the most common site is the right-sided colon. It is often diagnosed at an advanced stage with distant metastasis and has a worse prognosis compared with that of adenocarcinoma. Stage IV disease is linked to a mean survival period of 8.5 months (2). Considering the lack of treatment guidelines, therapeutic strategies for colorectal SCC are typically based on the management of adenocarcinoma. A 5-fluorouracil (FU)-based regimen is generally administered for the treatment of colorectal SCC. Copur et al. reported the case of a patient with SCC metastatic colorectal cancer (mCRC) who achieved an objective response after receiving cisplatin/etoposide/5-FU combination chemotherapy (3). Juturi et al. concluded that the combination of cisplatin, 5-FU, and leucovorin may be a treatment option for metastatic SCC of the colon (4). Considering the poor prognosis of colon SCC, the discovery of additional treatment strategies is urgently needed.

During the past decade, immunotherapy has rapidly developed and shown excellent antitumor efficacy against different types of cancer. The use of immune checkpoint inhibitors (ICIs) in the treatment of gastrointestinal tumors, particularly esophageal SCC, has resulted in great success. Nivolumab and pembrolizumab (anti-PD-1 monoclonal antibodies) were approved by the US Food and Drug Administration for the treatment of mCRC patients with microsatellite unstable–high (MSI-H) or deficient mismatch repair (dMMR) tumors. However, ICIs are mostly efficacious against dMMR/MSI-H CRC, while pMMR/MSS CRC is less susceptible to these agents (5). Considering only 5% in stage IV CRC patients are detected as the dMMR/MSI-H phenotype, most CRC patients are not able to benefit from ICIs alone (6). ICI monotherapy exerted a limited antitumor effect in CRC patients with pMMR/MSS tumors. Therefore, novel strategies (including immunotherapy) are currently under evaluation to improve treatment outcomes and the quality of life. It has already been proven that oxaliplatin, fluorouracil, and leucovorin (FOLFOX) can activate PD-1+ CD8 T cells to induce tumor infiltration, which triggers tumor adaptive immune resistance, supporting the utilization of ICIs and chemotherapies like FOLFOX (7). Based on these findings, the combination of ICIs and chemotherapy has been extensively investigated to improve the clinical outcome of pMMR/MSS mCRC. The AtezoTRIBE trial revealed that the addition of atezolizumab to oxaliplatin, fluorouracil, leucovorin, and irinotecan plus bevacizumab prolonged the PFS of patients with mCRC in the pMMR/MSS subgroup (8).

However, due to the rarity of the disease, there are few reported cases involving the use of immunotherapy against colon SCC. In view of the overexpression of PD-L1, we selected sintilimab and mFOLFOX6 as the preferred treatment options for this case. The present patient received PD-1 blockade combined with chemotherapy for primary pMMR/MSS SCC of the ascending colon with high PD-L1 expression. This combination treatment demonstrated excellent antitumor efficacy.

The frequency of the expression of PD-L1 is observed at approximately 24% in colorectal adenocarcinoma (9), and related cases are rarely reported in colorectal SCC. In a previous case of adenosquamous carcinoma with PD-L1 overexpression, PD-L1 expression was restricted to the SCC component (10). Squamous cancer cells may express higher levels of PD-L1 than adenocarcinoma cells in colorectal adenosquamous carcinoma samples. High PD-L1 expression can predict the response of various types of cancer to anti-PD-1/PD-L1 therapy. Nevertheless, PD-L1 expression has not been approved as a predictive biomarker for immunotherapy in CRC. Although the use of immunotherapy for pMMR/MSS CRC adenocarcinoma was initially not recommended, the treatment of pMMR/MSS colon SCC patients with high PD-L1 expression using ICIs was associated with robust and durable clinical outcomes. He et al. reported a case of rectosigmoid-junction SCC with high PD-L1 expression and pMMR/MSS tumors. The patient achieved a significant clinical benefit after combination treatment with sintilimab and capecitabine (11). In the present case, we treated a patient with colon SCC, high expression levels of PD-L1, and the pMMR/MSS phenotype using PD-1 blockade combined with chemotherapy. The patient achieved an excellent durable response. Thus, it appears that PD-L1 expression is a potential prognostic biomarker for PD-1 blockade therapy in patients with colorectal SCC.

There were no effective and standard regimens for colorectal SCC. The present case shows that combined immunotherapy is effective for the treatment of patients with colorectal SCC and high PD-L1 expression. It might be a good treatment regimen for patients with colon SCC. Sintilimab with the mFOLFOX6 regimen is applied for the first time to treat the primary SCC of the ascending colon with a high expression of PD-L1, the pMMR/MSS status, and the BRAF^V600E^ mutation. A significant antitumor efficacy was observed in the patient, suggesting that PD-1 blockade combined with chemotherapy may be an effective treatment for colorectal SCC patients. The level of PD-L1 expression may be a potential prognostic biomarker for the colorectal primary SCC.

Further randomized clinical trials with larger samples are warranted to validate the antitumor effect of PD-1 blockade combined with chemotherapy in patients with colorectal SCC, as well as to identify predictive biomarkers.

## Data availability statement

The original contributions presented in the study are included in the article/supplementary material. Further inquiries can be directed to the corresponding author.

## Ethics statement

The studies involving human participants were reviewed and approved by Weifang People’s Hospital. The patients/participants provided their written informed consent to participate in this study. Written informed consent was obtained from the individual(s) for the publication of any potentially identifiable images or data included in this article.

## Author contributions

YL wrote the manuscript. JD, PZ, WM, HX gathered the patient’s data. HX revised the manuscript. All authors contributed to the article and approved the submitted version.
